# How do mothers of adolescents with chronic pain experience their own quality of life?

**DOI:** 10.1186/s40359-020-00430-4

**Published:** 2020-06-16

**Authors:** S. Skarstein, A. K. Bergem, S. Helseth

**Affiliations:** Faculty of Health and Science, Oslo Metropolitan University, Postboks 4, St. Olavs plass, 0130 Oslo, Norway

**Keywords:** Adolescents, Chronic pain, Mothers, Quality of life, Non-prescript pain medicine, Over-the-counter analgesics

## Abstract

**Background:**

Chronic pain is a major health problem globally with severe personal and economic consequences. Maternal chronic pain is associated with their children’s pain. Family pain models and shared environmental aspects are important in the understanding of chronic pain among adolescents. Pain in itself impairs the quality of life (QoL). However, satisfaction in the aspects of health and functioning, social and economic, psychological, and family life will together constitute a person’s subjective experience of QoL. On this background, we considered it important to gain an understanding of the QoL of mothers who have children with chronic pain. We aimed to gain a broader understanding of the QoL in mothers of children with chronic pain and to investigate how they managed their children’s pain.

**Methods:**

This study had a qualitative design with face-to-face, in-depth interviews. The concept of QoL was used as a framework for developing a thematic, semi-structured interview guide. Eight mothers of adolescents with chronic pain (two boys and six girls) participated with signed consent.

**Results:**

Socio-economic difficulties and health complaints were common. Psychological stress, because of their children’s physical pain and other stressful experiences such as bullying, dominated everyday life. Lack of predictability and of responsible involvement from the fathers’ side increased the mothers’ burden considerably. Experiencing not being helped by others such as health professionals resulted in feelings of helplessness.

**Conclusions:**

These mothers had reduced QoL caused by their own health problems, concern for the child’s well-being and lack of social support, which affected the child’s upbringing and pain management. By improving these mothers’ QoL, family-based shared pain management strategies could help in health promotion, leading to a more positive QoL circle. Elements of family and cognitive therapy could be applied when supporting the mothers and children and improving their QoL.

## Background

Chronic pain is a major health problem world-wide, with consequences for individuals as well as for society as a whole [1, 2]. There are massive health care costs related to chronic pain. Only the costs of wage replacement and welfare programmes for those out of work because of pain are comparable [3]. Chronic pain in mothers is associated with chronic non-specific pain and chronic multi-site pain in adolescents and young adults [4]. The literature shows that as many as 15–35% of adolescents suffer from recurrent or chronic pain conditions, which affects their quality of life (QoL) negatively [5]. The higher the intensity and frequency of pain, the lower the self-reported QoL is, in adolescents of both genders [2]. Psychological functioning (e.g., feeling less at ease), physical status (a greater incidence of other somatic complaints), and functional status (more impediments to leisure and daily activities) were all impaired [2]. Chronic pain among adolescents had a negative impact on family and social life [2]. Mothers reported problems dealing with the stress of their children’s pain [2]. Research has shown correlations between children’s health and parents’ health-related quality of life (HRQOL) [6–9]. However, family structures influence this relationship, indicating that family pain models and shared environmental factors are important for the origin of chronic pain among adolescents [4]. Research has also supported the importance of social learning factors in influencing children’s pain experiences and it is possible that maternal attitudes influence adolescents’ QoL [10]. Maternal self-medication with non-prescription analgesics also influences the use of such medicines among their children, and such maternal behaviour can have an impact on their adolescents’ pain and their reports of pain [11].

Adolescents frequently use non-prescription pain medication to alleviate common ailments. Use of such medicines is associated with the experience of pain [12, 13] and this takes place in early age among adolescents [14]. Adolescents also have access to non-prescription medications at home [14, 15]. Pain among adolescents is common and affects daily activities such as school attendance, participation in hobbies, maintenance of social contacts, and sleep [4, 16]. Frequent use of over-the-counter (OTC) drugs might be how some adolescents deal with health problems [17]. From a long-term perspective, use of medicines early in life to alleviate pain and stress might prevent one from learning healthier ways of managing pain. Potentially it may lead to lifelong medicine use. If more effort is made on improving the life situations that adolescents perceive as painful and uncomfortable or in developing more expedient coping strategies, the need for OTC analgesics might be reduced [18].

An individual’s beliefs, expectations, and goals influence their experience of QoL, which is a subjective phenomenon and a normative concept. However, QoL is also a multidimensional concept that includes physical, psychological, social, and existential dimensions of life [19]. QoL is also defined as psychological well-being, implying that a person’s well-being is sensitive to the effects of life events [20]. Ferrans [21] defines QoL as “a person’s sense of well-being that stems from satisfaction or dissatisfaction with the areas of life that are important to him/her” [21, 22]. Further, satisfaction in health and functioning, social and economic, psychological (spiritual), and family will constitute a person’s subjective experience of QoL [21]. There is a lack of knowledge about QOL in mothers to adolescents with unspecific pain. On this background, we considered it important to gain an understanding of the QoL of mothers who have children with chronic pain.

### Aim

We aimed to gain understanding of the QoL in mothers of children with chronic pain and to investigate how they managed their children’s pain.

## Materials and methods

This study had a qualitative design with face-to-face, in-depth interviews with mothers of adolescents with chronic pain and frequent consumption of non-prescription analgesics.

In-depth interviews allow the interviewer to deeply explore the respondent’s feelings and perspectives on a subject. This results in rich background information that can shape further questions relevant to the topic. The key characteristics of in-depth interviews are the following: having open-ended questions, using a planned semi-structured format, to seek understanding and interpretation and recording responses [23].

### Settings and participants

This study was part of a larger study programme aiming to get broader knowledge and deeper understanding of frequent pain and use of non-prescription analgesics among adolescents [24–26].

For recruitment to this study, all adolescents in the 9th and 10th grades from 10 high schools in southern Norway were informed and invited to participate in the study. The included adolescents should have used non-prescription analgesics for pain daily over at least 4 weeks, within the past year, this without having acute or chronic illness requiring intake of non-prescription pain medication. The adolescents received verbal information from a researcher and received an information package at school, which they handed over to their parents. Possible participants contacted the researcher to be included, and if they met the inclusion criteria, time for an interview was scheduled.

Nineteen adolescents voluntarily contacted the researcher because they wanted to participate; all met the inclusion requirements and were included. Eight of them voluntarily recruited their parents. Eight mothers participated voluntarily with signed consent. The same researcher (the psychiatric nurse) conducted all interviews in a meeting room at the researcher’s workplace or at the adolescents’ schools. Interviews lasted about 45 min. Written consent was required and given in all interviews. The participants were informed both verbally and in writing of the possibility of withdrawing their consent at any time.

### Interview guide

Our research group consisted of four health professionals; one professor with backgrounds as both a general practitioner and pharmacist, two professors with background as public health nurses, and one associated professor with background as a psychiatric nurse. The research group had a collaboration with a team of five experienced school nurses from secondary schools. Together these health professionals used their professional knowledge and -experience, together with theory regarding the concept of QoL when developing themes for a thematic, semi-structured interview guide.

We wanted to get information on what the mothers’ thought was particularly important to share to shed light on their children’s pain. Family life, familial issues and relationships, social situations, reflections on efforts, and management of the children’s pain, as well as managing in general were the focus. The interview guide was designed with open-ended questions to gain broad and in-depth information, for example: “Tell me about your life” and “Tell me about your adolescent’s life”. On this basis, topics such as family life, social life, everyday life, coping and coping strategies, feelings, leisure-time activities, health, pain, and use of non-prescription analgesics were explored. As far as possible, the participants were encouraged to share their thoughts, reflections, and experiences.

Participants were recruited and interviewed until the research group was confident that no new themes had emerged, and saturation had been reached [27]. The transcribed material was organized and saved in the data management program NVivo, which supported the researcher during analysis [28–30].

### Analysis

In our analysis, we searched for meaningful units and interpretations in the text and subtext at both descriptive and interpretive levels [31]. The structure of the analysis of the interviews was done within the contexts of: *Self-understanding* of own statements that refers to how the participants reflect and understand their own expressions; *Common sense* that refers to how people in general think critically about the statement; *Main theme* that reference to the overall understanding and *Theory* that refers to a scientifically tested assumption of an expression as a phenomenon or in connection to nature (Table 1) [32].

**Table 1:** Example of the analyses of statements from interviews with mothers of adolescents who frequently used analgesics

Saying	Self-understanding	Common sense	Main themes	Theoretical perspective
*She complains mainly of headaches and a stiff neck. I compare this with my own experience with pain, which also began during puberty. I remember my mother’s history with pain and my grandmother’s as well. As far back as I know the women in my family have suffered from headaches. I have also been treated, treatment for headaches many, many times. The first time was when I was 11 years old. (018.0030)*	My child is trapped in the “legacy” of our family history, it repeats itself. I think there is probably nothing to do about this.	It seems to be a feeling of powerlessness and loneliness in life. A sense of lack of opportunities for pain relief. The mother organizes her everyday life from the perspective that she is the only caring and responsible adult.	**My child is just like me**	Significant degree of responsibility and high degree of loneliness seem to characterize the mothers’ thoughts and actions. These aspects both individually and through interaction, might impair QoL. They might underpin and reinforce each other.
*She grew up without a dad …*. *So, I was alone …*. *and I denied her [Contact with her dad, author’s note] for various reasons with alcohol. I didn’t trust him. I still don’t trust him. (812.0019)*	I cannot trust the father of my child. I am anxious that my child might not be well or get hurt.		**Parenting is a lonely project**	
… and suddenly it came out then what the problem was … She was actually sexually harassed badly in school. Moreover, it wasn’t just she that was harassed … and I’ve talked to school counsellors and everything at school. (812.0019)	I am the only person that really care about my child		**I am responsible**	
*I have chosen not to have a boyfriend; I can’t see any reason to … I’m not so stupid to believe that some guy would love my kids like I do. I’m not that dumb! (0018.0014)*	I downgrade my own needs for the good of the child.		**I downgrade my own needs**	

The researchers read the transcripts several times and organized the quotes. Potential meaningful sayings were noted. The researchers used common sense with a critical point of view to interpret and comment upon the participants sayings. When doubt or disagreement occurred, the researchers went back to the raw material and interpreted the meaning of the participant’s expressions. We searched for common meaningful units, patterns, or differences within the material. Stepwise, a broader understanding arose between the researchers. When agreement upon the meaningful units was reached, our findings were merged into main categories presented in the results. Theoretical understanding with dimensions of the theory on QoL was in the third context used to interpret the text, as described by Kvale and Brinkman [33].

In the results, representative quotes from the interviews are presented to give an accurate picture of the participant’s expressions. An English–Norwegian translator has translated the quotes into English and further back into Norwegian, this to ensure that the meaning was valid. To ensure confidentiality, the quotations are presented without any name, locality, gender, or age of the participant.

### Consent and ethical considerations

The Regional Committee for Medical and Health Research Ethics (REC) in Norway, approved the study in the autumn of 2012, and for this particular part of the study, in the autumn of 2018. Ethical principles from the Declaration of Helsinki were followed throughout the process [34].

The research group comprised a psychiatrist, a psychiatric nurse, and a public health nurse. The research group addressed the potential problems related to the researchers’ pre-understanding and discussed the benefits and challenges to reliability. We strove to be true to the participants’ expressions and discussed how our pre-understanding might affect our perception, and possibly our findings [35].

## Results

The mothers of two boys and six girls participated, a total of eight. Three were married, one was a widow, and four were single mothers with a variety of collaborations with the children’s fathers (Table 2). None of the mothers was working at the time of the interviews. In some of the families the child with pain was the only child, in others he or she had siblings. The married mothers had husbands with high academic skills, good financial positions, and high workloads.

**Table 2:** Some sociology-demographic characteristics of the participants

Mothers (*n* = 8)	
Married	3
Living without a partner	5
Employed, but on temporary sick leave	5 (3 of these were married)
Unemployed	3

The participants described parenthood as a lonely project where they seemed to have had troublesome health issues marked by pain of their own, lasting over a long time. The mothers took responsibility for their children’s well-being to a high degree. They were highly worried and concerned about their children’s pain and QoL.

Throughout the interviews, it also emerged that mothers had other burdens of care, for example, serious and long-term sick parents, close relationships with family members who struggled with mental challenges and substance abuse problems, as exemplified by this quote from one of the mothers who is married to a very highly positioned leader within health care:

*The father of my children is a periodic drinker. He is an alcoholic … but not too many know this. (p;47)*

Some told about their own childhood and upbringing under stressful conditions, like this story:

*‘There was always stress due to my father’s sickness, this was part of my childhood. From the time I was 11 or 12, he suffered from depression … depression with aggression. Never physically, but he had the need to have control all the time. Everything was dangerous. … He never received proper help. It was tough, and a few years ago I hit the wall … yeah, a fierce unpredictable anger … ” (p;31)*

Through analysing the eight interviews the following three main themes emerged: “My child is just like me”, “Parenting is a lonely project”, I am responsible”, and “I downgrade my own needs”.

### My child is just like me

The interviewed mothers were highly engaged in caregiving to alleviate their children’s pain. They were in some ways well informed about the adolescent’s everyday life, in particular physical and social stresses such as pain and bullying, while psychological stress, death of a beloved parent or divorce, and were rarely mentioned topics.

The mothers worried and tried to find ways to support their children. The mothers themselves struggled with pain of their own and linked the adolescents’ pain to their own experiences, as this quote illustrates:

*She complains mainly of headaches and a stiff neck. I compare this to my own experience with pain, which also began during puberty. I remember my mother’s history with pain and my grandmother’s as well. As far back as I know the women in my family have suffered from headaches. I have also been treated for headaches many, many times. The first time was when I was 11 years old. (p;30)*

Often mothers recommended their own coping strategies regarding pain relief for their children. When asked which of the parents the adolescents seemed most similar to, all the mothers mentioned themselves, often pointing out similarities in responsibility, caring attitudes, pain, and health issues. The following quote illustrates these findings:

*Actually, I look at her as a healthy and good girl, except she struggles with her self-perception. In many ways I see myself in her. I was also exposed to … . I didn’t have an eating disorder, but I did experience it. True enough, when I was a little older, I made myself throw up. Not a lot, just sometimes, when I felt guilty for over-eating and got a bad feeling. I always thought I was overweight, even when I was 20 kilos lighter. (p;39)*

### Parenting is a lonely project

The single mothers described how they had tried to share the responsibility for the child with others, such as the child’s father. In the end, the experience was that the fathers were neither able to help, nor willing to support and care. In some cases, the mothers had additional worries when their child spent time with the father, and the fathers were not mentioned as supportive or as partners to depend upon, as this quote illustrates:

*She grew up without a dad … . So, I was alone … . and I denied her [Contact with her dad, author’s note] for various reasons to do with alcohol. I didn’t trust him. I still don’t trust him. (;19).*

The married mothers told about their husbands’ heavy workload, as some had academic or business careers, mostly involving a lot of travelling. Several of the fathers in the single-parent families were mentioned as having their own problems, often related to drug or alcohol abuse. More than half of the single mothers described the fathers in ways that suggested they were perceived as additional burdens in the mothers’ everyday life, as these quotes point out:

*The agreement was that my child would visit his dad every other weekend. However, very often his dad would call on Thursday, right before my child was to leave, and cancel the visit … This led to my child not wanting to go … so, I would tell him he could stay home, and we would do something for fun … (p;36)*

*… and maybe since he is in the situation he is in now; he really does have enough with himself. He forgets things, including this. He’s showing signs of being a bit burnt out. (p;30)*

*No, the dad behaves like … The father is a user, even though he doesn’t admit it himself. (p;36)*

Implied in the mothers quotes we found an attitude towards the fathers as persons who are not to be relied upon. Geographical distance, obligations elsewhere, substance abuse, disruptive behaviour, and absence by death were some of the reasons given. This strengthened the mothers’ experiences of being the sole responsible adult, and of caring as being a lonely struggle.

It also appeared that the interviewed mothers had little support from their extended families, as this quote illustrates:

*However, it is clear that it has been very, very tiring, because I have not had any family that has helped me. Therefore, it is clear that it has been tiring. I never had any parents I could call to ask to come and help me … because my mother was sick. (p;14)*

In addition, these three quotes illustrate that the mothers experienced medical assistance and health professionals as not being very supportive or useful, and overall limited:

*In a way you are fighting with the health-care system, at times I feel that my child’s pain is not taken seriously … At least when compared with the pain my child is in, it’s not taken seriously (p;14)*

*I have somehow tried alternative therapy such as massage and yes naprapathy and chiropractors and all those who can offer treatment to the neck and body. (p:30)*

*I’d like to go to an acupuncturist. I would love to take my child too, but I can’t afford it. (p:36)*

### I am responsible

A sense of responsibility affected the mothers strongly in their thoughts, feelings, and actions. This extended to different levels, such as pain management, coping in everyday life, and in a sense for development of the child’s challenges in life in general. Several of the mothers told about their worries and feelings of helplessness because their children were victims of bullying, and how they had to take action to get help. These quotes express such concerns:

*… and suddenly it came out then what the problem was … She was actually sexually harassed badly in school. Moreover, it wasn’t just that she was the only one harassed … and I’ve talked to school counsellors and everything at school. The school counsellors said she had never experienced such a thing as in this school class, where the friendship groups among the girls at school were in a constant state of. But … it turned out that the boys also begat to harass the girls badly. (p;19)*

Several mothers said that during their children’s first years, they had worked hard both with education and in demanding work situations. Several reported that this might have caused situations where they did not fulfil the mothering role well enough. It seemed that some considered this as having had an adverse impact on the child’s upbringing, as this quote indicates:

*I was probably a bit affected by the fact that I wouldn’t let having a child stop me from living my life. It probably influenced the situation by not being there enough … and now I suddenly realize that she is 15 … . and I think, shit that went very fast … If only I had just been able to take it easy. (p;30)*

The mothers reported allocating resources, attention, time, and money trying to help their adolescents in getting pain relief. Massaging their children, helping with schoolwork, making appointments with medical doctors, health professionals, and therapists, making sure the adolescents’ food was healthy, providing motivational support for a good balance between rest and activities, and having non-prescription analgesics available were examples of such efforts.

The mothers had strong opinions about analgesics as a remedy not to be used unnecessarily, and they took upon themselves the role of gatekeeper. They all described their administration of analgesics as restrictive, but necessary, as these quotes illustrate:

*First of all, I am against painkillers unless they are really needed. So, he has had Ibuprofen and Aspirin before bedtime. And we try to make it through the day without them. (p:17)*

*… if she has a headache then she gets a tablet. Actually, I have been very restrictive of taking anything myself and am careful not to take them too often. (p:19)*

As mentioned above, some of the single mothers talked about their worries when their child visited with their fathers because of the father’s mental disturbances and alcohol and drug abuse. Nevertheless, they continued to have contact with the fathers. Several told they supported their former partner with money and care, but at the same time, they expressed anger, especially in cases when the fathers had new partners. However, the mothers pointed out that financial support was for the children’s benefit during visitations. In many ways, it seemed as though the mothers had ambivalent relationships with their children’s fathers. Some highlighted positive sides of their former partner, as this quote illustrates:

*My ex-husband had problems when we were together … Well not actually problems … He was drug addicted … but happily addicted, not depressively drug addicted … Do you understand? He had a responsible, full-time job and was very popular – a role model. So, there was nothing like … there was no fuss, if you know what I mean. (p:36)*

### I downgrade my own needs

Most mothers were out of work, either permanently or for a period of time. Among the reasons for not being employed were loss of a job, sick leave because of illnesses, and withdrawal from work to take care of the child or sick parents. Most reported that they missed being part of a work life, but that it was too hard to meet both job requirements and demands in everyday life. Some mothers said helpful treatment was more costly than they could afford. The single parents reported financial struggles at the same time as wanting to give their children the same opportunities as their peers, as this mother said:

*I’m looking forward to seeing the performance with her, but I’m not over excited either … because actually I’m broke and can’t afford the trip. But I’m getting a little money back on my tax refund … so, I’ll try to take a little from here and a little from there. (p:19)*

Some of the single mothers told of avoiding getting a new partner, some because a new partner would not care enough about their child, as this quote illustrates:

*I have chosen not to have a boyfriend. I can’t see any reason to … I’m not so stupid to believe that some guy would love my kids like I do. I’m not that dumb! (p:14)*

Others hid the fact of having a partner so as not to make it difficult for their child. Most reported little or no contact with the father’s extended family and commented that these relationships were commonly conflicted.

## Discussion

The mothers’ efforts regarding their children and pain were substantial. Attention, time, and money were allocated for seeking solutions to reduce both their own and their children’s stresses and strains. Social structures in Norway have changed; many parents are single, with grandparents, aunts, and uncles often living scattered [36]. Having responsibility for children and teenagers without family support can be demanding. Further, most healthy adults are in the workforce and have limited time to assist other family members. As in the stories told by the interviewed mothers, this might be demanding and experienced as a lonely responsibility, especially when the child is struggling with persistent pain without a medical diagnosis. Signals that could indicate that something is wrong with the child might be frightening and in turn provoke worry and anxiety for the mothers. Lack of control over the child’s health and upbringing and at the same time feeling responsible for caring might cause a stressful situation. Through the interviews, it also seemed that the mothers not only felt responsible for their child, but also were involved in caring for others, such as their own parents and their children’s father. This aspect of caring for the children’s fathers, was especially mentioned by the single mothers as it produced mixed feelings. Research during the last decade has continued to show that, compared with children with continuously married parents, children with divorced parents score lower on average on a variety of emotional, behavioural, social, health-related, and academic measures [37]. The accumulation of welfare problems both increases the chance of a relationship being dissolved and of single people remaining single [38]. When the mothers—in addition to being single parents—also had conflicts in their relationships with their children’s fathers, it is reasonable to believe that this affected their QoL. Furthermore, this might have impaired their adolescent children’s QoL, as good family relationships are important for this [39]. A negative spiral, in which mothers and children had reduced experiences of feeling good in several aspects of QoL, could interact and reinforce the negative feelings over time.

The family is the setting for children’s upbringing and represents fundamental social and economic resources for the adults. Studies have demonstrated the importance of impaired family background and family relationships for the development of many types of mental illness [40, 41]. It is nevertheless worth noting that there may also be significant stresses in two-parent families with good finances. In addition, in the families in this study, the mothers took primary responsibility for their children’s pain and pain management.

Research shows that feeling good, being satisfied with oneself, having an overall positive attitude, and a positive self-image are important for good QoL. The interviewed mothers had stressful everyday lives, with pain and worries. Some told about adverse childhood experiences, which resulted in negative psychological reactions in adult life. Stress might be a precipitant to pain, while family conflicts, adverse family experiences, loneliness, and feeling like an outsider can be intervening variables. Imbalance between the demands that are placed on a person and the ability to manage those demands might cause stress [42]. The mothers described their children’s pain and complaints as similar to their own. This might be a way of making the children’s pain recognizable and understandable. However, through childhood and upbringing the mothers had learned how to express themselves to others. According to Bowlby and Ainsworth’s attachment theory [43], an individual’s attitude is shaped early in life and in a close relationship with “important others” [44, 45]. It can therefore be that, through upbringing, health challenges and pain have become meaningful and important in interactions with others. Such patterns might be transferred to their children and become a communication structure between mother and child [26]. QoL is a subjective phenomenon and a normative concept based on the individual’s beliefs, expectations, and goals.

The interviewed mothers had struggled with health complaints of their own for a long period. Pain and reduced daily functions were common and this seemed to affect their QoL negatively. Research has shown an association between maternal and child pain, where the presence of multiple pain sites in the mother consistently predicted the presence of site-specific pains and multiple pains in the child. The risk for a child to get back pain, headache, and stomach pain is significantly increased if mothers have pain at similar sites [46]. A study by Frare et al. (2002) identified a relationship between headache severity, child coping, and QoL in children [47]. Frequency and duration of pain have a significant impact on a child’s QoL; nevertheless, family daily routines significantly influence both the child’s coping ability and thereby their QoL. The study also showed that the better children were at coping with pain, the better their QoL [47]. This means that if the daily routine within the family works well, the child copes better with pain and his or her perceived QoL is better. However, it is then also a reason to believe that QoL in the mothers will affect their adolescent children’s QoL.

The lack of a definitive medical diagnosis or better treatment options for pain both for themselves and for their child seemed to push the mothers in our study towards a continuous search for helpful treatment and the right therapists. This takes resources such as money, time, energy, and attention; nevertheless, the mothers’ willingness to prioritize their children’s well-being was very high and sometimes almost without limits. As tools to help the children, the mothers made sure that non-prescription analgesics were available and used. The interviewed mothers stated that their children’s use of painkillers was as recommended. Further, they conveyed a restrictive attitude and were concerned with the dangers of high consumption. According to Chambers et al. [10], maternal behaviour might have a direct impact on their children’s subjective reports of pain, and this could underpin the importance of the social learning aspects of the mothers’ influence on children’s pain experiences [10].

The interviewed mothers were out of work: some because they were employed, some because of illnesses, and some found it too difficult to handle job expectations in addition to their own and their family members’ health challenges in everyday life. None of the single mothers was in the workforce. Being outside a working life leads to loss of arenas where the interviewed mothers could receive feedback and support. Meaningful employment is one criterion for experiencing good QoL [48]. Furthermore, experiences of meaningful activity, coping, and growth can be lost. Not having a job in addition often means having a poorer economic situation than those in employment, especially in a single-parent family [36]. Several of the interviewed mothers were single parents and had limited economic resources. In Norway, both situations can lead to feelings of inferiority [36]. Such socio-economic aspects might therefore affect the mothers’ QoL negatively, both by reducing the possibility for themselves and their children to participate in desired activities, and by being dependent on others. This might in turn have had an impact on the experience of their own values and opportunities.

The interviewed mothers had pain, care burdens, concerns about issues of both social and financial character, and worries for their children’s well-being. These factors would have affected their QoL negatively. They also experienced the support from health professionals as being limited. Systemic family therapy based on cognitive models has been found to be an effective intervention for children and adolescents with, for example, anxiety, depression, or substance abuse. Such intervention might also be helpful for supporting these families [49–51].

### Strengths and limitations

The research group included participants from different disciplines who analysed the findings, and this might have given broader perspectives and increased insight than a more restricted group. A board-certified psychiatric nurse who is also the main author conducted all interviews. Her clinical experience and professional background could have promoted obtaining in-depth information. The participants were of the same gender as the interviewer and this might have lessened barriers of communication across genders.

One limitation of the study was the small sample size. Therefore, we should be careful not to transfer the experiences from this study to all mothers of adolescents with chronic pain frequently using non-prescription analgesics. Another limitation of this study is that we did not include the fathers of the adolescents. Including fathers could have given other perspectives and broader knowledge. We emphasize that results of qualitative studies cannot be generalized, but information revealed might contribute to a deeper and broader understanding. However, if our findings are recognized and the reader acknowledges their usefulness, they could be considered transferable within the framework of qualitative research [33, 52].

## Conclusions

These mothers of adolescents with chronic pain had reduced QoL. By increasing the mothers’ QoL, aspects of the adolescents’ QoL can also increase. More research is needed both to design a guide for professional mapping and to give health support to increase the QoL for mothers of adolescents with chronic pain. Through this study, we have identified possible perspectives that might lead to novel approaches to identify, guide, and support families with adolescents with chronic pain. Individual tasks, family matters, and socio-economic perspectives must be considered both in mapping and when it comes to interventions. Elements from family therapy and cognitive therapy may be applied when supporting mothers of adolescents with chronic pain to gain a better QoL. Family therapy may be an opportunity to address family dynamics and social inheritance.

## Data Availability

The material is stored at Faculty of Health and Science, Oslo Metropolitan University until December 2020. The datasets generated and/or analysed during the current study are not publicly available due to participant confidentiality. Anonymous parts of the material can be available by contacting the corresponding author.

## Abbreviations

QoL: Quality of life
OTC: Over-the-counter
HRQOL: Health-related quality of life
REC: The Norwegian Regional Committees for Medical and Health Research Ethics
P: Participant
